# Using ISU-GAN for unsupervised small sample defect detection

**DOI:** 10.1038/s41598-022-15855-7

**Published:** 2022-07-08

**Authors:** Yijing Guo, Linwei Zhong, Yi Qiu, Huawei Wang, Fengqiang Gao, Zongheng Wen, Choujun Zhan

**Affiliations:** 1 School of Information Science and Technology, Xiamen University Tan Kah Kee College, Zhangzhou, 363105 China; 2 School of Informatics, Xiamen University, Xiamen, 361005 China; 3 School of Aerospace Engineering, Xiamen University, Xiamen, 361005 China

**Keywords:** Information technology, Computer science

## Abstract

Surface defect detection is a vital process in industrial production and a significant research direction in computer vision. Although today’s deep learning defect detection methods based on computer vision can achieve high detection accuracy, they are mainly based on supervised learning. They require many defect samples to train the model, which is not compatible with the current situation that industrial defect sample is difficult to obtain and costly to label. So we propose a new unsupervised small sample defect detection model-ISU-GAN, which is based on the CycleGAN architecture. A skip connection, SE module, and Involution module are added to the Generator, enabling the feature extraction capability of the model to be significantly improved. Moreover, we propose an SSIM-based defect segmentation method that applies to GAN-based defect detection and can accurately extract defect contours without the need for redundant noise reduction post-processing. Experiments on the DAGM2007 dataset show that the unsupervised ISU-GAN can achieve higher detection accuracy and finer defect profiles with less than 1/3 of the unlabelled training data than the supervised model with the full training set. Relative to the supervised segmentation models UNet and ResUNet++ with more training samples, our model improves the detection accuracy by 2.84% and 0.41% respectively and the F1 score by 0.025 and 0.0012 respectively. In addition, the predicted profile obtained using our method is closer to the real profile than other models used for comparison.

## Introduction

Products may have surface defects in the actual industrial production process due to machine errors, worker errors, and production process problems. Surface defects not only affect the aesthetics and performance of the product, resulting in lower user satisfaction but may also be a safety hazard, posing a threat to the life and property of the user. Hence, surface defect detection is an essential part of industrial production.

For a long time, the process of industrial surface defect has relied on manual work, which is not only time-consuming and laborious but also very subjective, which cannot meet the needs of industrial production with high efficiency and precision. Therefore, automated defect detection technology based on computer vision has been a more popular research direction. Currently, automated defect detection methods based on machine vision mainly include traditional methods and deep learning methods.

### Traditional methods

Traditional methods rely on the structural information of the image to detect defects. It usually requires human efforts to design the corresponding detection algorithm based on the characteristics of the defect and the actual application scenario. Current traditional defect detection methods based on machine vision mainly include Gabor filtering^1^, improved local binary pattern algorithm (MB-LBP)^2^, improved Sobel algorithm^3^, etc. Most traditional vision methods rely heavily on specific defect features and are difficult to achieve end-to-end detection. The time and economic cost of designing different inspection algorithms for different defects by hand are very high and require a large number of people with strong expertise, which is challenging to meet industrial production’s efficiency and cost requirements. Furthermore, in practice, detection algorithms based on the characteristics of defects seen by the human eye are susceptible to interference from changes in the external environment, making it difficult to achieve satisfactory robustness.

### Deep learning methods

With the advent of deep learning, various algorithms based on Convolutional Neural Networks (CNNs) have achieved surprising results in many subfields of machine vision. Compared to traditional defect detection methods, deep learning methods mostly eliminate the need to manually model defect features and enable end-to-end detection. They also have the advantages of high detection accuracy, fast convergence, and robustness.

Specifically, standard deep learning methods for defect detection include target detection methods, semantic segmentation methods and novel generative adversarial network-based detection methods.

#### Object detection

Object detection is a fundamental task in the field of machine vision. The aim of which is to detect the position or category of a specific object in a given image. Specifically in the application of defect detection, the area where the defect is located is used as the object to be detected. Standard detection networks in the field of defect detection are SSD^4^, YOLO^5^, Faster R-CNN^6^, etc. In defect detection area, Object Detection models usually perform well in speed and accuracy. However, the inability to extract defect profiles is still a major disadvantage because fine profile can helps to explore the causes of defects in industry.

#### Semantic segmentation

Unlike object-level classification for Object Detection, Semantic Segmentation pursues pixel-level classification. In contrast to Object Detection, which only needs to give object-level prediction frames, Semantic Segmentation requires a classification prediction for each pixel of the image. Thus, the use of semantic segmentation networks for defect detection locates the defect and segments the actual contour of the defect. The current mainstream segmentation networks in defect detection are Mask R-CNN^7^, UNet^8^, SegNet^9^, etc. Semantic Segmentation models can extract the contours of defects, but since it classifies on a per-pixel basis, it requires a large number of training samples and pixel-wise annotation.

#### Generative adversarial networks

Many machine vision-based defect detection techniques still have two significant challenges for practical industrial applications. Firstly, training samples containing defects are challenging to obtain. Secondly, manual labeling of training samples is costly. In this case, the Generative Adversarial Networks (GAN)^10^ offers a new way of thinking in defect detection with its powerful data generation capabilities. It creatively pits two functionally different networks (Generator G and Discriminator D) against each other. They are trained against each other to improve their respective performance, resulting in a Generator that can generate fake-to-true data. The defect detection network used in this paper is based on one of the variants of GAN-CycleGAN^11^. CycleGAN is essentially two mirror-symmetric GANs, which can learn two different distributions of samples between mapping relationships, and is widely used in computer vision fields such as image translation and style migration.

Since the training of GAN networks is usually unsupervised, it saves much of the cost associated with dataset annotation, which is a significant advantage in its practical application.

## Related works

### General deep learning defect detection

In recent years, there has been a proliferation of deep learning methods for defect detection, with many novel models achieving good detection results on specific datasets. For example, Lee et al.^12^ proposed a real-time decision-making method for steel surface defect detection based on CNN and class activation maps. Mei et al.^13^ used Denoising Autoencoder Networks with Gaussian pyramids to reconstruct defects and combined with multi-scale fusion to detect surface defect in fabrics with good results. Zhong et al.^14^ proposed PVANET++ based on Faster R-CNN, which associates the low-level feature map with the high-level feature map to form a new superexpression map for proposal extraction, applied in detecting defects in railway cotter pins. Tabernik et al.^15^ designed a two-stage detection model based on the segmentation network and discriminative network. It extracted fine defect profiles on the KolektorSDD dataset. Huang et al.^16^ proposed an improved MCue module with UNet to generate saliency images for detecting magnetic tile surface defect. Li et al.^17^ proposed an improved UNet with Dense Block module and summation skip connection to detect concrete surface cracks, and the method achieved an average pixel accuracy of 91.59% and an average IoU of 84.53% on the concrete defect dataset. Inspired by UNET and DenseNet, the DefectSegNet proposed by Roberts et al.^18^ adopts skip connection within and between blocks, which shows high pixel accuracy in a high-quality steel defect datasets.

Current surface defect detection models based on general deep learning can achieve high detection accuracy and real-time requirements, but they mostly require a large number of negative samples and labels for training, which is costly and difficult to implement in industrial applications.

### GAN-based defect detection

Using GAN for surface defect detection is a relatively novel approach, first seen in AnoGAN^19^ proposed by Schlegl et al. in 2017. AnoGAN learns a streaming distribution of positive samples in the potential space during the training phase, while the testing phase iteratively finds the nearest vector in this space and then compares the generator output with the original map to find the anomalous region. As the iterative optimization in the training phase was too time-consuming, so the authors proposed an improved version of f-AnoGAN with encoder structure^20^ in 2019. f-AnoGAN alleviates the problem of huge time consumption to a certain extent. Other similarly improved versions include Zenati et al.^21^ and Akcay et al.^22^. Niu et al.^23^ used the original CycleGAN to fix and detect defects. They used much more samples to train the network and it is difficult to obtain stable detection performance in the case of complex defect backgrounds.

In response to the difficulty of obtaining defect samples in industrial applications, Di et al.^24^ combined convolutional self-encoder (CAE) and semi-supervised generative adversarial network (SGAN) to propose a semi-supervised CAE-SGAN to obtain better detection results with less training of hot-rolled sheet images. He et al.^25^ proposed a fusion algorithm based on cDCGAN and ResNet to generate pseudo-labels for unlabelled samples and used it to train a defect detection model, which achieved good results on the NEU-CLS dataset. Zhao et al.^26^ proposed a positive sample-based detection method which used a Defect Generation Module to create defects for the positive samples and then trained a DCGAN to repair the defects. But how to generate defects close to the true distribution is a more difficult problem.

Although current GAN-based defect detection methods can be semi-supervised or unsupervised, they still only perform well on simple uniform textured surfaces. GAN networks that can be applied to complex industrial inspection environments need further research.

### Our method

To address the common problems of high annotation cost and difficulty in obtaining training data for deep learning defect detection, we designed an unsupervised ISU-GAN model and an SSIM-based defect extraction method. ISU is an abbreviation of Involution-SE-U, which means a U-shaped structured network using the Involution operator and SE operator. ISU-GAN is essentially an improved version of CycleGAN. The differences from the original CycleGAN network structure include: 1. The generator adopts a UNet-like structure to reduce the possible loss of defective features during the encoding-decoding process of the input image; 2. the SE operator is used for the feature maps of the critical layers to suppress the less important channels; 3. the Involution operator is used for the feature maps obtained by downsampling to meet the demand for different visual capabilities of defective and non-defective regions.

In the training phase, we want to learn to obtain generators that map positive samples (defect-free samples) and negative samples (defective samples) to each other. The defect repair network maps negative samples to positive samples and the defect manufacturing network maps positive samples to negative samples. In the testing phase. We input the test image into the defect repair network in the testing phase. We then use the Structural Similarity Algorithm (SSIM)^27^ to compare the original image and the repair image to obtain an SSIM score map with the same resolution as the original image. We finally use the OTSU algorithm^28^ to extract the contours of the defects adaptively.

Our method achieves an average accuracy of 98.43% and an F1 score of 0.9792 on the DAGM2007 dataset using only a small number of training samples. It can segment very accurate defect profiles. We also validate the superiority of our ISU-GAN network structure over other commonly used defect detection models and the effectiveness of its main modules through comparative and ablation experiments.

In general, the innovation of our work mainly includes the following two aspects.

#### Defect detection network

We propose a new GAN defect detection network, ISU-GAN, converging quickly and achieving excellent detection accuracy with a small training dataset.

#### Defect segmentation method

We propose an SSIM-based defect segmentation method that applies to GAN-based defect detection. Without labels required, our method can accurately extract defect contours with the absence of redundant noise reduction post-processing.

**Figure 1:**
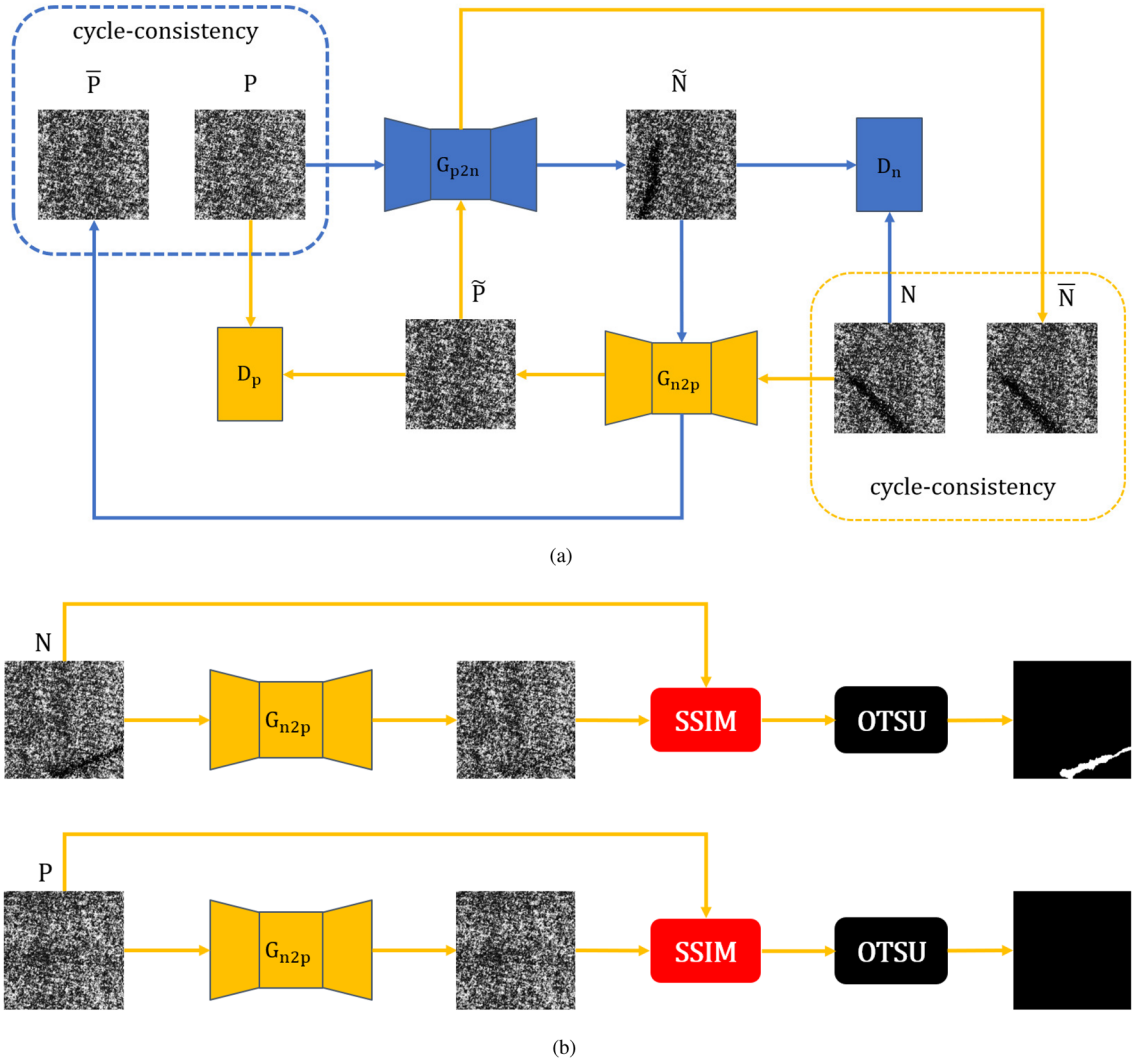
Principle of our detection method. (**a**) Training stage. (**b**) Test stage. $G_{n2p}$ and $G_{p2n}$ are positive and negative sample generators respectively, $D_p$ and $D_n$ are positive and negative sample discriminators respectively. SSIM is the Structural Similarity Algorithm, and OTSU is the OTSU Adaptive Threshold Segmentation Algorithm.

## Methodology

In that section, we describe the principle of the defect detection method proposed in this paper and the model structure of ISU-GAN. In the training phase, we train ISU-GAN to learn the mapping relationship between negative and positive samples. ISU-GAN is based on the CycleGAN architecture and consists of two cooperating GANs, as shown in Fig. 1. The solid orange line indicates $GAN_P$ and the solid blue line indicates $GAN_N$, which are the GANs for repairing defects and generating defects, respectively.

The first adversarial network $GAN_P$ consists of a Generator $G_{n2p}$ and a Discriminator $D_p$. The input to $G_{n2p}$ is the negative sample set *N* in the training dataset, which repairs the defective image regions in *N* and generates pseudo-positive samples $\widetilde{P}$ that do not contain defects. The input to the discriminator $D_p$ is the true sample *P* and the pseudo-positive sample $\widetilde{P}$, whose role is to distinguish *P* from $\widetilde{P}$. Correspondingly, another adversarial network $GAN_N$ consists of a generator $G_{p2n}$ and a discriminator $D_n$. The input to $G_{p2n}$ is the positive sample set *P* in the training dataset, which serves to add defects to the images in *P* and generate pseudo-negative samples $\widetilde{N}$ that contain defects. The input to the discriminator $D_n$ is the true negative sample *N* and the pseudo-negative sample $\widetilde{N}$, whose role is to distinguish *N* from $\widetilde{N}$.

Based on the cycle consistency criterion of CycleGAN, it is necessary to input $\widetilde{P}$ into $G_{p2n}$ to generate quadratic pseudo-negative samples $\overline{N}$. We expect $\overline{N}$ and *N* to be as similar as possible, i.e. $n\approx G_{p2n}(G_{n2p}(n)), n\in N$. Correspondingly, $\widetilde{N}$ is input into $G_{n2p}$ to generate a quadratic pseudo-positive sample $\overline{P}$, $p\approx G_{n2p}(G_{p2n}(p)), p\in P$.

In the test phase, the test dataset *X* (containing positive and negative samples) is fed into the defect repair generator $G_{n2p}$ obtained from training. For any sample $x\in X$, the SSIM algorithm is used to compare *x* and $G_{n2p}(x)$ to obtain the SSIM score map with the same resolution as *x* (the higher the score means the higher the region’s similarity). Then the OTSU adaptive threshold segmentation algorithm is used to segment the SSIM score map to determine whether there are defects in *x* and extract the possible defect contours.

### Network structure

#### Generator

**Figure 2:**
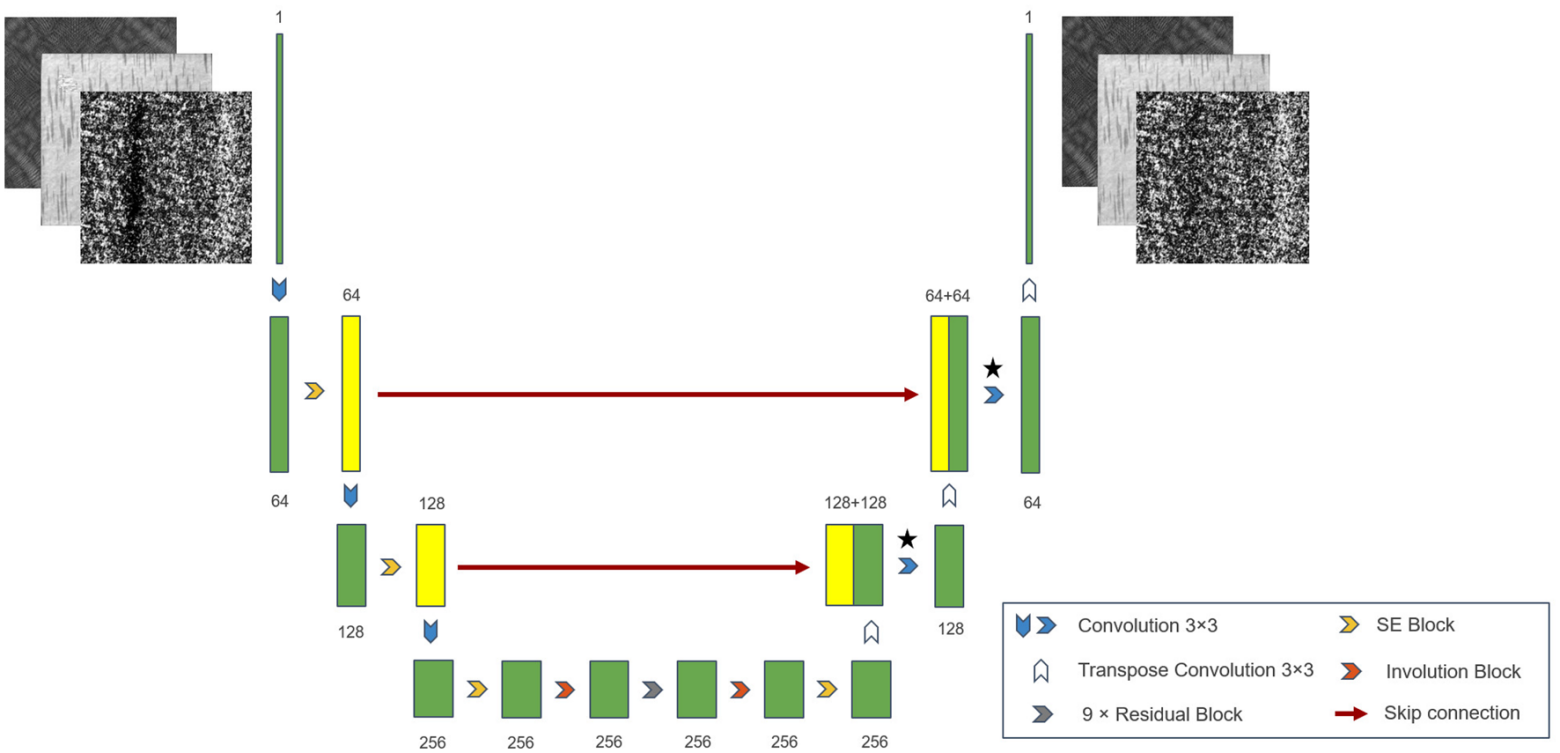
With a UNet-like architecture, the Generator network structure contains three convolutional downsampling layers and three transposed convolutional upsampling layers. The network aggregates information from the lower feature maps via skip connections, with an intermediate layer containing a Squeeze Excitation (SE) module and an Involution module to enhance feature extraction. *Inspired by StyleGANv2^29^, we remove the IN layer $\bigstar$ to eliminate artifacts that may appear in the generated image.

The Generator is based on the Encoder-Decoder design guidelines and has a general structure similar to UNet, as shown in Fig. 2. After the image is input to the Generator, it is first downsampled by three $3 \times 3$ convolutional layers to obtain a 256-channel feature map, which is then passed through the SE module to filter the channels of the feature map for importance. Its purpose is to take full advantage of the channel-independent properties of the next Involution module to focus on the more critical channels. Nine consecutive residual blocks follow the Involution layer to improve the convergence of the model. Further on are the symmetrically designed Involution and SE modules, and an upsampling layer implemented by three $4 \times 4$ transposed convolutions. In particular, to reduce feature loss from the downsampling–upsampling operation, we use a skip connection to aggregate information from the shallow and deep feature maps. So we filter the 64-channel and 256-channel feature maps from the downsampling operation by the SE module, then concatenate them with the feature maps corresponding to the same number of channels from the upsampling operation, and use a $3 \times 3$ convolutional layer to restore the channel count to its original state.

In the Generator structure, all convolutional layers except at $\bigstar$ carry Instance Norm and ReLU.

#### Discriminator

The Discriminator uses the PatchGAN structure^30^, containing only four superficial $4 \times 4$ convolutional layers. The input image is first transformed into a 512-channel feature map by passing through three convolutional layers with a multiplicative number of filters, and then downscaled to a single-channel feature map X by the action of a convolutional layer with a filter number of 1. Each pixel on X represents the discriminator’s score of the corresponding location region of the input image. Compared to conventional discriminators, the Discriminator of the PatchGAN structure can discriminate each patch of the input image differently, enabling the extraction of local image features, which is conducive to improving the detail quality of the generated image.

In the Discriminator structure, all convolutional layers come with Instance Norm and LeakyReLU with slope 0.2. LeakyReLU is used instead of ReLU to alleviate the gradient vanishing problem during training.

### Skip connection

To reduce the loss of image detail features due to the downsampling-upsampling process, we performed a skip connection between the 64-channel and 128-channel intermediate feature maps, see Fig. 2. The skip connection in ISU-GAN is to connect the shallow feature map to the deep feature map in the channel dimension (using a Reflection pad to adjust to the exact resolution if the two feature maps have different resolutions). Then A convolution of 3 $\times$ 3 is used to restore the feature map with double the number of channels to the original number of channels. In contrast to conventional skip connection, the shallow feature map is rescaled for channel importance before channel-connection, using the SE Block. The benefit of adding the SE module to the skip connection is that it provides a better aggregation of the essential features of the shallow feature maps, allowing the model to extract defect profiles with enhanced power.

### Squeeze-and-excitation block

**Figure 3:**
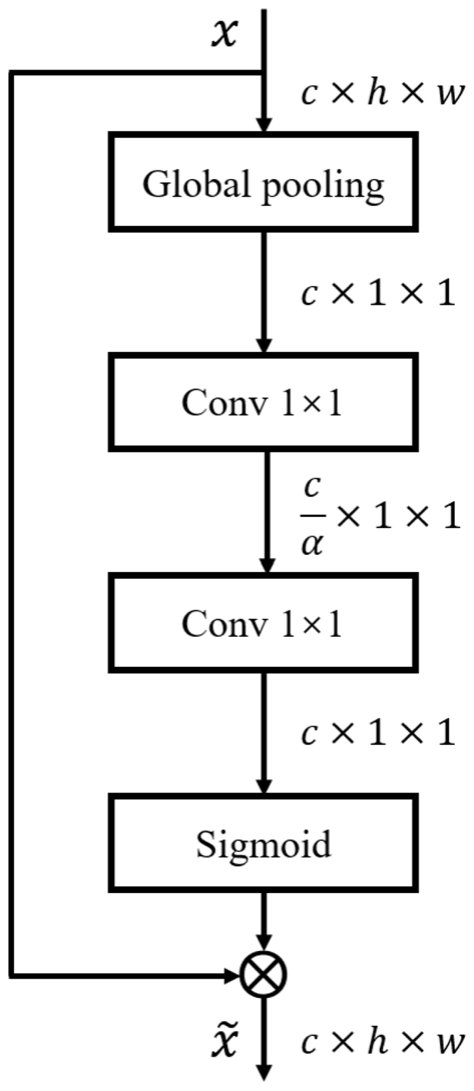
Structure of SE module. $\alpha$ is the channel downsampling ratio and $\otimes$ denotes the tensor multiplication operation.

Squeeze-and-excitation block is a module proposed in Ref.^31^ that learns the relationship between individual feature channels to obtain the weight of each channel, thus rescaling the importance of all channels. It allows the model to focus more on channels with important information and suppress non-important ones. The flow chart of SE Block is shown in Fig. 3.

#### Squeeze

The Squeeze operation performs feature squeezing on each channel of the feature map, converting the two-dimensional map into a real number that aggregates all the features on the channel. In this case, global average pooling is used to implement the squeeze operation, as in Eq. (1).

1 $$\begin{aligned} y=\frac{\sum _{i=1}^{H}\sum _{j=1}^{W} x_{i,j}}{H\times W}. \end{aligned}$$

#### Excitation

The Excitation operation aims to learn the interrelationships between the different channels of the feature map and evaluate each channel’s importance. Two successive $1 \times 1$ convolutions with a filter number of $\frac{c}{\alpha }$ and *c*, where $\alpha$ is the channel downscaling factor to reduce the network parameters. After two convolutions and ReLU activation, the $c \times 1 \times 1$ vector representing the importance of each channel is then mapped between 0 and 1 using the Sigmoid function. The process is as in Eq. (2).

2 $$\begin{aligned} z=Sigmoid\left( W_2 ReLU \left( W_1 y \right) \right) . \end{aligned}$$

Finally, the channel importance vector *z* obtained from learning is multiplied by the original feature map *x* to obtain the rescaled feature map $\widetilde{x}$, i.e. $\widetilde{x} = z\cdot x$. The SE Block has four applications in our Generator network (as shown in the red part of Fig. 1), two before the skip connection and two in the middle layer of 256 channels.

### Involution block

The traditional convolution operator has two main properties: space-independence and channel-specificity. While its space-independence makes convolution efficiency guaranteed, it deprives the convolution kernel of the ability to adapt to different patterns in different regions. The problem of channel redundancy within the convolution has not been solved even in many well-known CNN networks.

At the recent CVPR2021, the Involution module^32^ was proposed to address this problem. The involution operator, which has space-specificity and channel-independence in contrast to convolution, uses the kernel generation function $\phi$ to generate different convolution kernels for different location regions of an image. The Involution operator gives the network different visual patterns based on different spatial locations.

The shape of the Involution kernel *H* depends on the size of the input feature map *x*, and the kernel generation function generates *H* based on specific pixels.

3 $$\begin{aligned} H=\phi \left( x_{i,j} \right) =W_2 \sigma \left( W_1 x_{i,j} \right) , \end{aligned}$$

where $W_1$ and $W_2$ represent linear transformations and $\sigma$ denotes BN and ReLU. $W_1$ reduces the representation of location-specific pixels of $c \times 1 \times 1$ to $\frac{c}{r} \times 1 \times 1$ (r represents the reduction ratio), which $W_2$ then changes to $G \times k \times k$. G is the number of channels in each group, and all channels in the group share the parameters of the kernel *H*, which is typically set to 16. Finally, the generated kernel *H* performs a single-step convolution operation on a specific pixel region.

For surface defect detection, the use of the Involution module meets the need for different visual capabilities in different areas of the image (defective and non-defective regions), allowing the model to extract more realistic defect contours.

### Structural similarity

Structural similarity (SSIM) is an algorithm that measures the similarity of two images, taking into account the image’s brightness, contrast, and structural characteristics. SSIM measures these differences through the luminance comparison function *l*(*x*, *y*), the contrast comparison function *c*(*x*, *y*) and the structural comparison function *s*(*x*, *y*), respectively.

4 $$\begin{aligned} l\left( x,y\right)&=\frac{2\mu _x\mu _y+C_1}{\mu _x^2+\mu _y^2+C_1},\end{aligned}$$

5 $$\begin{aligned} c\left( x,y\right)&=\frac{2\sigma _{xy}+C_2}{\sigma _x^2+\sigma _y^2+C_2 },\end{aligned}$$

6 $$\begin{aligned} s\left( x,y\right)&=\frac{\sigma _{xy}+C_3}{\sigma _x\sigma _y+C_3}, \end{aligned}$$

where $\mu _x$, $\sigma _x$, and $\sigma _{xy}$ denote the mean of x, the variance of x, and the covariance of x and y, respectively. To simplify the form, let $C_3=C_2/2$. The SSIM exponential function is expressed as Eq. 7.

7 $$\begin{aligned} \begin{aligned} SSIM\left( x,y\right) =l\left( x,y\right) \cdot \left( x,y\right) \cdot s\left( x,y\right) =\frac{\left( 2\mu _x\mu _y+C_1\right) \left( {2\sigma }_x\sigma _y+C_2\right) }{\left( \mu _x^2+\mu _y^2+C_1\right) \left( \sigma _x^2+\sigma _y^2+C_2\right) }. \end{aligned} \end{aligned}$$

It is better to find the SSIM index locally than globally in image quality assessment. Thus the mean, variance, and covariance in the above equations are calculated in the local area within the sliding window. The final global SSIM score is the average of the scores of all the local regions within the sliding window. The size of SSIM window is a hyperparameter. Through experimental comparison, we set it to 9. The SSIM algorithm can be used not only to measure the similarity of two images but also as a loss measure during model training, called SSIM loss. SSIM loss has the advantage of fast training convergence, so this paper uses SSIM loss in the pre-training phase to reduce the required training time.

### Loss function

In ISU-GAN, we use three loss functions types: Adversarial Loss $L_{GAN}$, Cycle Consistency Loss $L_{cycle}$ and Identity Loss $L_{identity}$.

#### Adversarial loss

$L_{GAN}$ is divided into $L_{GAN\_G}$ and $L_{GAN\_D}$ in terms of specific implementations, which represent the optimization targets of the generator G and the discriminator D, respectively. The adversarial loss is measured using L2 loss, as shown in Eqs. (1) and (2), where **0** and **1** represent the full 0 tensor and the full 1 tensor, respectively. G wants the generated fake samples to deceive D, i.e. the fake input samples make the discriminator output as close to 1 as possible. On the contrary, D wants to distinguish between real and fake samples as much as possible. Thus when the input is a real sample, D wants its output to be as close to 1 as possible. While for a fake sample, the output is as close to 0 as possible.

8 $$\begin{aligned} \begin{aligned} L_{GAN\_G}=&\sum _{p\in P}\left( D_n\left( G_{p2n}\left( p\right) \right) -\mathbf {1}\right) ^2+\sum _{n\in N}\left( D_p\left( G_{n2p}\left( n\right) \right) -\mathbf {1}\right) ^2, \end{aligned} \end{aligned}$$

9 $$\begin{aligned} \begin{aligned} L_{GAN\_D}=\sum _{p\in P}\left[ {\left( p-\mathbf {1}\right) ^2+\left( D_n\left( G_{p2n}\left( p\right) \right) -\mathbf {0}\right) }^2\right] + \sum _{n\in N}\left[ \left( n-\mathbf {1}\right) ^2+\left( D_p\left( G_{n2p}\left( n\right) \right) -\mathbf {0}\right) ^2\right] . \end{aligned} \end{aligned}$$

#### Cycle consistency loss

We want the samples obtained from the real samples after sequentially going through a forward mapping and a reverse mapping to be as consistent as possible with the original samples to improve the stability of the generated model, i.e. $G_{n2p}(G_{p2n}(p)) \approx p$ and $G_{p2n}(G_{n2p}(n)) \approx n$. We use the Cycle Consistency Loss $L_{cycle}$ to measure this similarity. In particular, to combine the advantages of fast convergence of SSIM loss and high detail fidelity of L1 loss, we use a loss function replacement strategy for $L_{cycle}$. We first train k epochs using SSIM loss to allow accelerated convergence, and then replace it with L1 loss to optimize the detail of the generated images, as shown in Eq. (10), where we empirically set k to 10.

10 $$\begin{aligned} L_{cycle}=\left\{ \begin{aligned}&\sum _{p\in P}\left[ 1-SSIM\left( p,G_{n2p}\left( G_{p2n}\left( p\right) \right) \right) \right] +\sum _{n\in N}\left[ 1-SSIM\left( n,G_{p2n}\left( G_{n2p}\left( n\right) \right) \right) \right] ,&e<k\\&\sum _{p\in P}\vert p-G_{n2p}\left( G_{p2n}\left( p\right) \right) \vert +\sum _{n\in N}\vert n-G_{p2n}\left( G_{n2p}\left( n\right) \right) \vert ,&e\ge k\\ \end{aligned} \right. \end{aligned}$$

#### Identity loss

To reduce the probability of predicting a positive sample as a negative sample, we want the defect repair generator $G_{n2p}$ not to change the positive sample too much. To avoid unnecessary interference noise, we expect p to be as similar as possible to $G_{n2p}(p)$. We use the Identity Loss $L_{identity}$ to measure this degree of dissimilarity.$L_{identity}$ uses the same loss function replacement strategy as $L_{cycle}$, as shown in Eq. (7).

11 $$\begin{aligned} L_{identity}=\left\{ \begin{aligned}&\sum _{p\in P}\left[ 1-SSIM\left( p,G_{n2p}\left( p\right) \right) \right] ,&e<k\\&\sum _{p\in P}\vert p-G_{n2p}\left( p\right) \vert ,&e\ge k\\ \end{aligned} \right. \end{aligned}.$$

## Experiment

### Dataset

**Figure 4:**
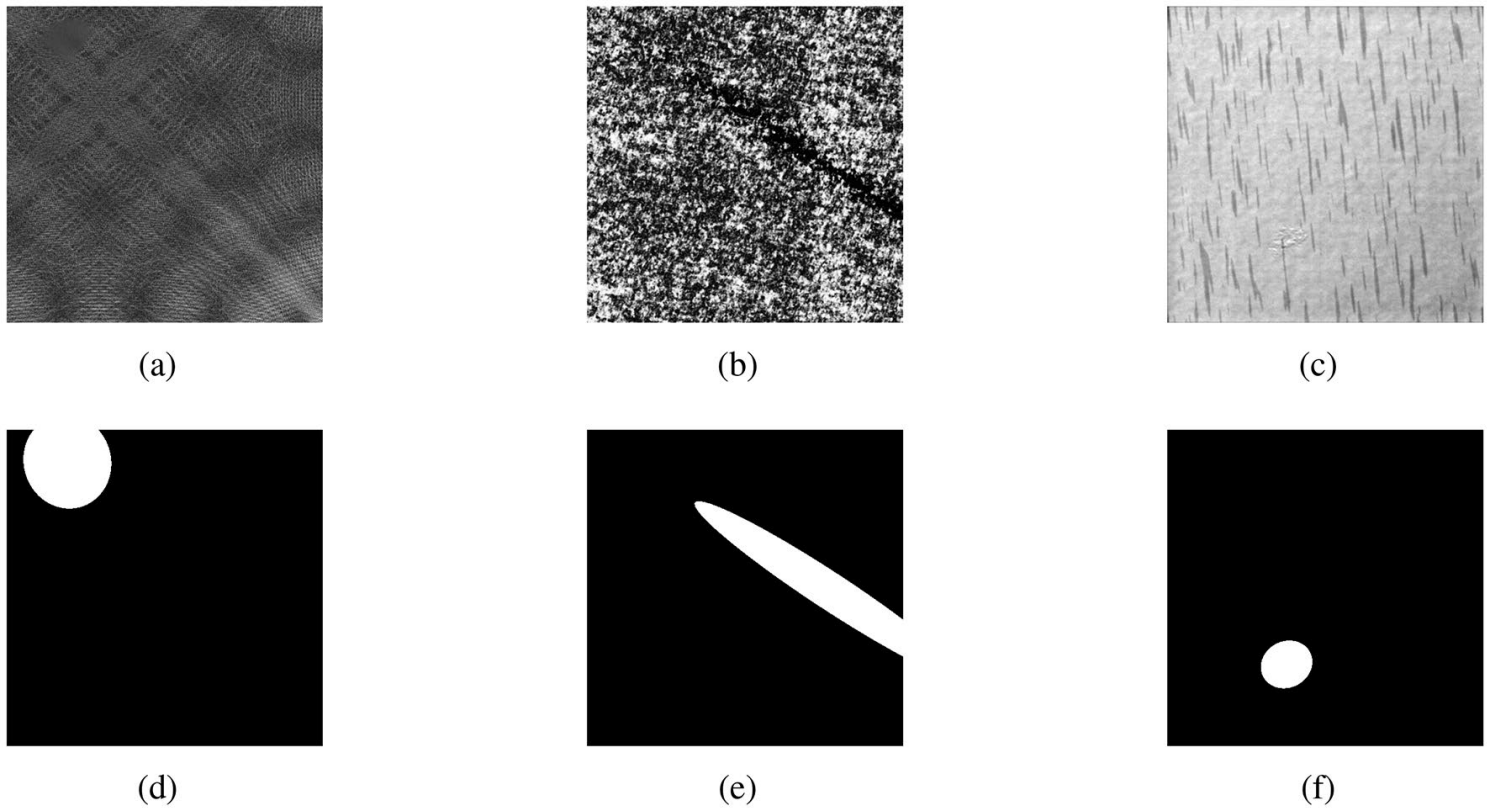
The three selected categories of defective samples and their labels. (**a–c**) Are Class 1, Class 6, and Class 7 respectively, and (**d**–**f**) are their corresponding labels.

DAGM2007^33^ is a well-known dataset for industrial weakly supervised defect detection, which contains ten artificially produced texture defects. This dataset is downloaded from https://hci.iwr.uni-heidelberg.de/node/3616. Each class is divided into a training set and a test set. All images in DAGM are grey-scale images of 512 $\times$ 512, where the defect images are labeled with weak supervision. We selected three of these representative classes (as in Table 1) for our experiments. Class 1 has more diverse surface texture. Class 6 has messier surface texture. Class 7 has sliver defects. We chose these three classes to test the robustness of ISU-GAN for diverse textures, messy textures, and sliver defects respectively. The defect images for the three used classes are shown in Fig. 4.

**Table 1:** Division of the DAGM2007 dataset.

		Class 1$^{1}$	Class 6	Class 7	Sum
Ours	Train	400P + 80N	100P + 50N	100P + 50N	600P + 180N
	Test	504P + 71N	508P + 67N	1000P + 150N	2102P + 288N
Other$^{2}$	Train	575P + 80N	575P + 83N	1150P + 150N	2300P + 313N
	Test	504P + 71N	508P + 67N	1000P + 150N	2102P + 288N

$^1$Due to the wider texture variety of Class 1 images, our ISU-GAN needs to use a higher number of Class 1 images to train the model.

$^2$For the other models used for comparison, to obtain a stable result, we used the entire training set (including the label) officially divided by DAGM.

### Evaluation metrics

In the comparison experiments in this paper, we use Accuracy (Acc) and F1-score to compare the defect detection effectiveness of the different models. In the ablation studies, we use F1-score and MSE to examine the impact of different modules on network performance.

Here we define TN: predicted defective sample and actually defective sample; FN: predicted defective sample but actually non-defective sample; TP: predicted non-defective sample and actually non-defective sample; FP: predicted non-defective sample but actually defective sample.

#### Accuracy

Accuracy is defined as the proportion of all correctly predicted samples, as in Eq. (2).

12 $$\begin{aligned} Accuracy=\frac{TN+TP}{TN+FN+TP+FP} \times 100\%. \end{aligned}$$

#### F1-score

F1-score is a statistically significant measure of the accuracy of a dichotomous model, defined as the summed average of Precision and Recall:

13 $$\begin{aligned}&Precision = \frac{TN}{TN+FN},\end{aligned}$$

14 $$\begin{aligned}&Recall=\frac{TN}{TN+FP},\end{aligned}$$

15 $$\begin{aligned}&F1=\frac{2\times Precision \times Recall}{Precision+Recall}. \end{aligned}$$

#### MSE

In our ablation studies, we use Mean Square Error (MSE) to measure the similarity between the pseudo-positive samples restored by the defect repair generator and the original positive samples. Its lower value indicates that the reconstructed image is closer to the original one in detail. We do not use negative samples when calculating the MSE because the better the repair is for the defective region, the higher the MSE will be. For this paper, the MSE is calculated as the average of all positive samples.

16 $$\begin{aligned} MSE=\frac{1}{N_P}\sum _{p\in P}\left( p-G_{n2p}\left( p\right) \right) ^2. \end{aligned}$$

### Implementation details

The experimental environment used in this paper is as follows: CPU: Intel(R) Xeon(R) CPU E5-2620 v4 @ 2.10GHz, GPU: GeForce GTX 1080Ti, Memory: 128G, Python: 3.6.13, Pytorch: 1.7.1.

To improve the convergence of the model, we resize the input image from 512 $\times$ 512 to 256 $\times$ 256, and the interpolation method used is bicubic^34^. To improve the robustness of the model, the batch size is set to 1, and all input images are performed with equal probability in one of the following three operations: (1) keeping constant, (2) flipping horizontally, and (3) flipping vertically. Our network was trained from the beginning for all experiments, using the optimizer Adam^35^, with an initial learning rate of 0.0002 and a training epoch of 100. In the comparison experiments section, we will compare the performance of ISU-GAN with commonly used defect detection segmentation models (UNet, ResUNet++) and the classic GAN networks (original CycleGAN, DCGAN) for defect detection and segmentation. In the ablation studied section, we will compare the impact of each ISU-GAN module on the network performance.

### Comparison experiments

**Figure 5:**
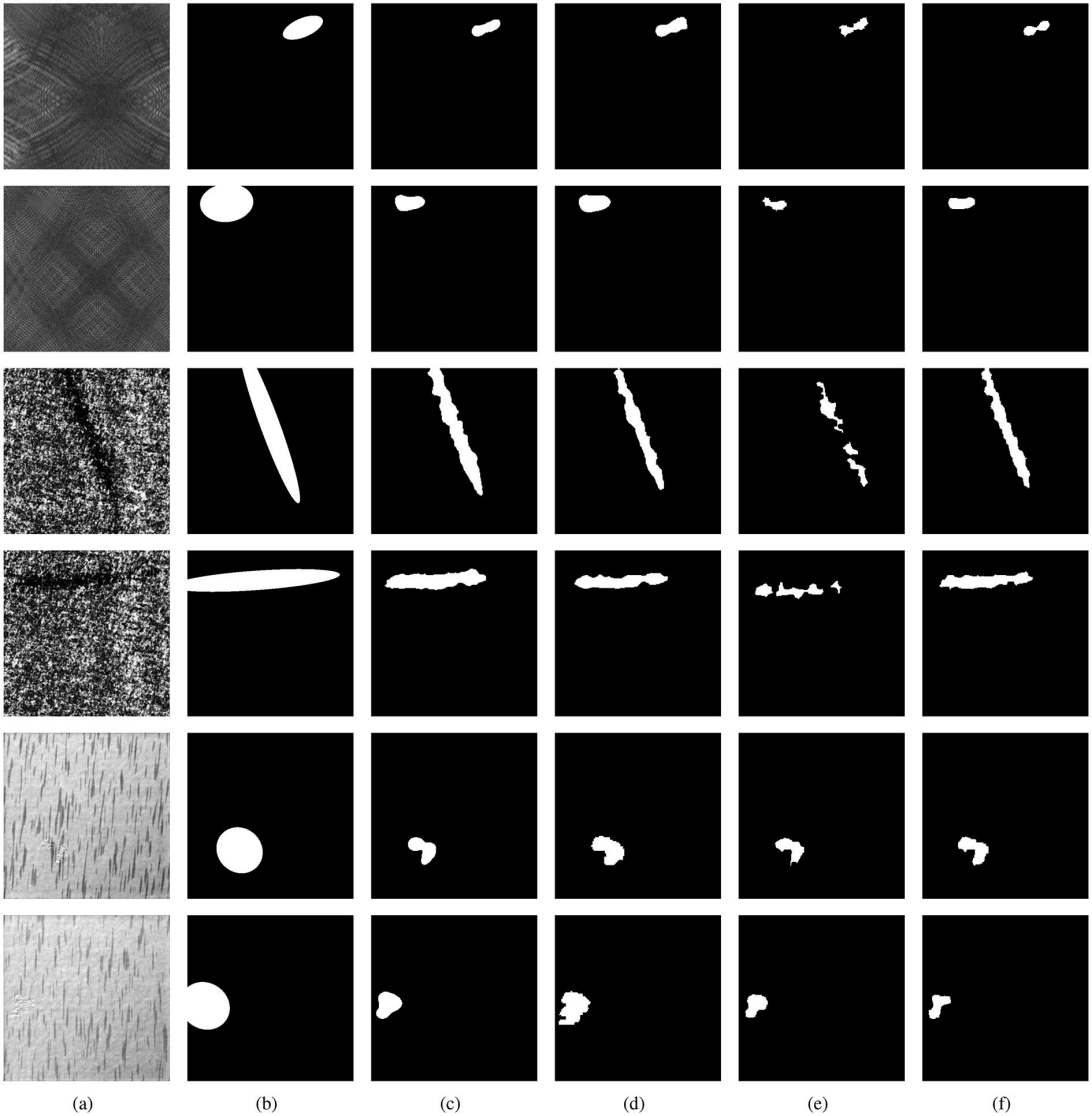
Comparison of defect extraction results. (**a**) Original image, (**b**) label, (**c**) UNet, (**d**) ResUNet++, (**e**) DCGAN, (**f**) ISU-GAN.

In this section, we compare the defect detection and segmentation performance of our ISU-GAN with some models. The models used for comparison include the classical GAN networks CycleGAN and DCGAN, the commonly used semantic segmentation models UNet and its improved version ResUNet++. UNet is one of the classical models of semantic segmentation, often used as a benchmark model for various segmentation tasks, and it is also widely used in the field of defect detection^17,18^. ResUNet++ is a relatively new member of the UNet family, which combines the advantages of ResNet and UNet, and introduces SE blocks to show more powerful image segmentation capabilities. In section related works, We mentioned that CycleGAN^23^ and DCGAN^26^ have been implemented for the DAGM dataset with good results, so we chose these GAN for comparison. The experiment results in the test stage are shown in Fig. 5 and Table 2.

From the experimental results, it can be seen that despite using less than one-third of the training data of the other models and without labels, ISU-GAN still shows an improvement of more than 2.5% in the average two metrics compared to UNet. ResUNet++, an improved version of UNet, performs markedly better than UNet in all categories, but its Acc and F1 are lower than ISU-GAN by about 0.4% and 0.1%. In contrast, when comparing the detection results of CycleGAN and DCGAN, ISU-GAN has significantly improved in all categories of data, with over 1.5% and 3.0% improvement on average. By comparing the test data of each model, it can be verified that our method is effective.

It is worth mentioning that ISU-GAN performs significantly worse than ResUNet++ on Class 1 and is on the lower level of all Classes. The possible reason is that the wide variety of background textures in Class 1 makes it harder for our model to find the positive and negative sample mapping relationships that we expect.

**Table 2:** Comparing Accuracy and F1-score in ISU-GAN and other segmentation models.

	Class1		Class6		Class7		Average	
	Acc $(\%)$	F1	Acc $(\%)$	F1	Acc $(\%)$	F1	Acc $(\%)$	F1
UNet^8^	93.74	0.9342	94.78	0.9454	98.26	0.9831	95.59	0.9542
ResUNet++^36^	**97.21**	**0.9655**	98.61	0.9848	98.35	0.9836	98.02	0.9780
CycleGAN^23^	96.0	0.9420	97.39	0.9710	97.22	0.9714	96.87	0.9615
DCGAN^26^	92.87	0.9043	96.17	0.9412	97.39	0.9538	95.48	0.9331
ISU-GAN (Ours)	96.52	0.9524	**98.78**	**0.9853**	**100.0**	**1.0**	**98.43**	**0.9792**

$^{1}$The GAN Discriminators for the experiments use the Markov Discriminator^30^.

Significant values are in bold.

**Figure 6:**
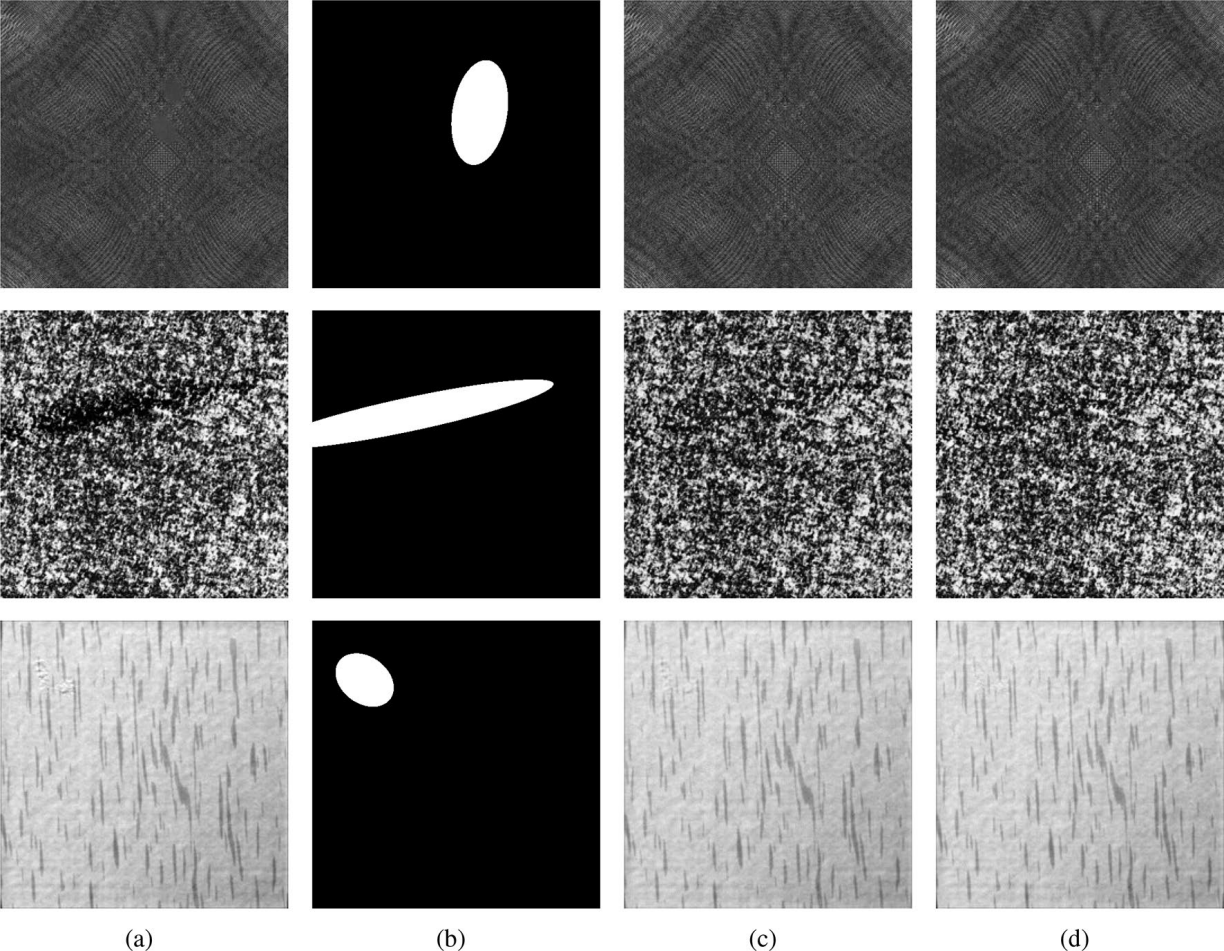
Comparison of defect repair results. (**a**) Original image, (**b**) label, (**c**) CycleGAN, (**d**) ISU-GAN.

As can be seen from Fig. 5, even without using labels during training, our model is more finely and accurately segmented for defects than Supervised Learning-based UNet and ResUNet++, which will benefit workers in the manufacturing industry to determine the type of defects. With the same unsupervised training, DCGAN method needs to manually create defects for the images, which is more tedious. While our method omits this procedure and has significantly better results. We also compare the defect repair results of ISU-GAN and CycleGAN, see Fig. 6. It can be observed that the repair map generated by ISU-GAN is closer to the original image in detail, especially the texture at the edges is smoother and more realistic.

### Ablation studies

Ablation studies were set up to investigate the impact of three crucial modules (skip connection, Involution, SE) in the generator structure of ISU-GAN on the effectiveness of defect detection. The Generator models compared in the ablation experiment are: 1. the original CycleGAN (default); 2. using only one of the three modules; 3. using all three modules (ISU-GAN).

The dataset and hyperparameters used for the ablation experiments are the same as section comparison experiments, and all submodels use the method proposed in section methodology to detect defects. The results of the experiments are shown in Table 3.

On average, the improvement of skip connection for the model lies mainly in the significant reduction of MSE, but the improvement of F1-score is not apparent. In contrast, the Involution Block improves the F1-score significantly but also increases the MSE noticeably, while the SE Block optimizes both values to a lesser extent. For the ISU-GAN with all three modules, we can see that it achieves the best results in both average values, and the improvement is significant compared to the original CycleGAN. It indicates that the ISU-GAN model structure is reasonable and practical.

**Table 3:** Comparing the performance of different modules in different classes.

	Class 1		Class 6		Class 7		Average	
	F1	MSE	F1	MSE	F1	MSE	F1	MSE
Default (CycleGAN)	0.9420	23.50	0.9710	44.19	0.9714	21.99	0.9615	29.89
Skip	**0.9589**	**19.17**	0.9504	10.31	0.9834	10.08	0.9642	13.18
Inv	0.9458	38.36	**0.9925**	74.23	0.9906	35.49	0.9763	49.36
SE	0.9412	21.34	0.9680	41.65	0.9888	23.51	0.9660	28.83
All (ISU-GAN)	0.9524	19.40	0.9853	**8.95**	**1.0**	**9.03**	**0.9792**	**12.46**

Significant values are in bold.

## Conclusion

From the results of this paper, our proposed defect detection model ISU-GAN and the associated defect extraction method can perform well under unsupervised conditions with a small number of training samples. ISU-GAN innovatively uses skip connection, SE block and Involution Block in the generator to obtain better defect feature characterization. Furthermore, the SSIM-based defect extraction method can extract more accurate defect profiles.

Through comparison experiments, we show that ISU-GAN can achieve a better defect detection effect even if the training conditions are much weaker than UNet and ResUNet++. Through ablation studies, we show the impact of the three main modules of ISU-GAN on the network performance and verify the effectiveness of the ISU-GAN structure.

In section comparison experiments, we mentioned that ISU-GAN performs significantly worse than other classes due to difficulty mapping positive and negative samples in data sets with richer texture types. According to this problem, we will further optimize the network structure to obtain a more robust performance in the subsequent work.

## Data Availability

Datasets used in this study are available to download at: Datasets used in this study are available to download at: https://hci.iwr.uni-heidelberg.de/node/3616.

## References

[1] Liu YB, Xiao ZT, Zhang F, Wu J (2011). Fabric defect detection method based on gabor filters. Adv. Mater. Res..

[2] Liu Y, Xu K, Xu J (2019). An improved mb-lbp defect recognition approach for the surface of steel plates. Appl. Sci..

[3] Shi T, Kong J-Y, Wang X-D, Liu Z, Zheng G (2016). Improved sobel algorithm for defect detection of rail surfaces with enhanced efficiency and accuracy. J. Central South Univ..

[4] Liu, W. *et al.* Ssd: Single shot multibox detector. In *European conference on computer vision*, 21–37 (Springer, 2016).

[5] Redmon, J., Divvala, S., Girshick, R. & Farhadi, A. You only look once: Unified, real-time object detection. In *Proc. IEEE Conference on Computer Vision And Pattern Recognition*, 779–788 (2016).

[6] Ren S, He K, Girshick R, Sun J (2015). Faster r-cnn: Towards real-time object detection with region proposal networks. Adv. Neural. Inf. Process. Syst..

[7] He, K., Gkioxari, G., Dollár, P. & Girshick, R. Mask r-cnn. In *Proc. IEEE International Conference on Computer Vision*, 2961–2969 (2017).

[8] Ronneberger, O., Fischer, P. & Brox, T. U-net: Convolutional networks for biomedical image segmentation. In *International Conference on Medical Image Computing and Computer-Assisted Intervention*, 234–241 (Springer, 2015).

[9] Badrinarayanan V, Kendall A, Cipolla R (2017). Segnet: A deep convolutional encoder-decoder architecture for image segmentation. IEEE Trans. Pattern Anal. Mach. Intell..

[10] Goodfellow, I. *et al.* Generative adversarial nets. *Adv. Neural Inf. Process. Syst.* **27** (2014).

[11] Zhu, J.-Y., Park, T., Isola, P. & Efros, A. A. Unpaired image-to-image translation using cycle-consistent adversarial networks. In *Proc. IEEE International Conference on Computer Vision*, 2223–2232 (2017).

[12] Lee SY, Tama BA, Moon SJ, Lee S (2019). Steel surface defect diagnostics using deep convolutional neural network and class activation map. Appl. Sci..

[13] Mei S, Wang Y, Wen G (2018). Automatic fabric defect detection with a multi-scale convolutional denoising autoencoder network model. Sensors.

[14] Zhong J, Liu Z, Han Z, Han Y, Zhang W (2018). A cnn-based defect inspection method for catenary split pins in high-speed railway. IEEE Trans. Instrum. Meas..

[15] Tabernik D, Šela S, Skvarč J, Skočaj D (2020). Segmentation-based deep-learning approach for surface-defect detection. J. Intell. Manuf..

[16] Huang Y, Qiu C, Yuan K (2020). Surface defect saliency of magnetictile. Vis. Comput..

[17] Li S, Zhao X, Zhou G (2019). Automatic pixel-level multiple damage detection of concrete structure using fully convolutional network. Comput.-Aided Civil Infrastruct. Eng..

[18] Roberts G (2019). Deep learning for semantic segmentation of defects in advanced stem images of steels. Sci. Rep..

[19] Schlegl, T., Seeböck, P., Waldstein, S. M., Schmidt-Erfurth, U. & Langs, G. Unsupervised anomaly detection with generative adversarial networks to guide marker discovery. In *International Conference on Information Processing in Medical Imaging*, 146–157 (Springer, 2017).

[20] Schlegl T, Seeböck P, Waldstein SM, Langs G, Schmidt-Erfurth U (2019). f-anogan: Fast unsupervised anomaly detection with generative adversarial networks. Med. Image Anal..

[21] Zenati, H., Foo, C. S., Lecouat, B., Manek, G. & Chandrasekhar, V. R. Efficient gan-based anomaly detection. Preprint at http://arxiv.org/abs/1802.06222 (2018).

[22] Akcay, S., Atapour-Abarghouei, A. & Breckon, T. P. Ganomaly: Semi-supervised anomaly detection via adversarial training. In *Asian Conference on Computer Vision*, 622–637 (Springer, 2018).

[23] Niu, S., Lin, H., Niu, T., Li, B. & Wang, X. Defectgan: Weakly-supervised defect detection using generative adversarial network. In *2019 IEEE 15th International Conference on Automation Science and Engineering (CASE)*, 127–132 (IEEE, 2019).

[24] Di H, Ke X, Peng Z, Dongdong Z (2019). Surface defect classification of steels with a new semi-supervised learning method. Opt. Lasers Eng..

[25] He Y, Song K, Dong H, Yan Y (2019). Semi-supervised defect classification of steel surface based on multi-training and generative adversarial network. Opt. Lasers Eng..

[26] Zhao, Z., Li, B., Dong, R. & Zhao, P. A surface defect detection method based on positive samples. In *Pacific Rim International Conference on Artificial Intelligence*, 473–481 (Springer, 2018).

[27] Wang Z, Bovik AC, Sheikh HR, Simoncelli EP (2004). Image quality assessment: From error visibility to structural similarity. IEEE Trans. Image Process..

[28] Otsu N (1979). A threshold selection method from gray-level histograms. IEEE Trans. Syst. Man Cybern..

[29] Karras, T. *et al.* Analyzing and improving the image quality of stylegan. In *Proc. IEEE/CVF Conference on Computer Vision and Pattern Recognition*, 8110–8119 (2020).

[30] Isola, P., Zhu, J.-Y., Zhou, T. & Efros, A. A. Image-to-image translation with conditional adversarial networks. In *Proc. IEEE Conference on Computer Vision and Pattern Recognition*, 1125–1134 (2017).

[31] Hu, J., Shen, L. & Sun,G. Squeeze-and-excitation networks. In *Proc. IEEE Conference on Computer Vision and Pattern Recognition*, 7132–7141 (2018).

[32] Li, D. *et al.* Involution: Inverting the inherence of convolution for visual recognition. In *Proc. IEEE/CVF Conference on Computer Vision and Pattern Recognition*, 12321–12330 (2021).

[33] Wieler, M. & Hahn, T. *DAGM Symposium in Weakly Supervised Learning for Industrial Optical Inspection* (2007).

[34] Keys R (1981). Cubic convolution interpolation for digital image processing. IEEE Trans. Acoust. Speech Signal Process..

[35] Kingma, D. P. & Ba, J. Adam: A method for stochastic optimization. In *International Conference on Learning Representations* (2015).

[36] Jha, D. *et al.* Resunet++: An advanced architecture for medical image segmentation. In *International Symposium on Multimedia* (2019).

